# Analyzing the Impacts of Off-Road Vehicle (ORV) Trails on Watershed Processes in Wrangell-St. Elias National Park and Preserve, Alaska

**DOI:** 10.1007/s00267-012-9811-z

**Published:** 2012-02-12

**Authors:** Christopher D. Arp, Trey Simmons

**Affiliations:** 1 Alaska Science Center, U.S. Geological Survey, Anchorage, AK 99508 USA; 2 Present Address: Water and Environmental Research Center, University of Alaska Fairbanks, Fairbanks, AK 99775 USA; 3 Central Alaska Inventory and Monitoring Network, National Park Service, Fairbanks, AK 99709 USA

**Keywords:** Boreal wetlands, Channel initiation, Headwater streams, Land-use disturbance, National parks, Off-road vehicles (ORV), Permafrost soils, Recreational trails

## Abstract

Trails created by off-road vehicles (ORV) in boreal lowlands are known to cause local impacts, such as denuded vegetation, soil erosion, and permafrost thaw, but impacts on stream and watershed processes are less certain. In Wrangell-St. Elias National Park and Preserve (WRST), Alaska, ORV trails have caused local resource damage in intermountain lowlands with permafrost soils and abundant wetlands and there is a need to know whether these impacts are more extensive. Comparison of aerial photography from 1957, 1981, and 2004 coupled with ground surveys in 2009 reveal an increase in trail length and number and show an upslope expansion of a trail system around points of stream channel initiation. We hypothesized that these impacts could also cause premature initiation and headward expansion of channels because of lowered soil resistance and greater runoff accumulation as trails migrate upslope. Soil monitoring showed earlier and deeper thaw of the active layer in and adjacent to trails compared to reference sites. Several rainfall-runoff events during the summer of 2009 showed increased and sustained flow accumulation below trail crossings and channel shear forces sufficient to cause headward erosion of silt and peat soils. These observations of trail evolution relative to stream and wetland crossings together with process studies suggest that ORV trails are altering watershed processes. These changes in watershed processes appear to result in increasing drainage density and may also alter downstream flow regimes, water quality, and aquatic habitat. Addressing local land-use disturbances in boreal and arctic parklands with permafrost soils, such as WRST, where responses to climate change may be causing concurrent shifts in watershed processes, represents an important challenge facing resource managers.

## Introduction

Off-road travel across boreal lowlands is challenging in the summer because of expansive mosaics of lakes, muskeg, bogs, and forest that occur atop permafrost soils with poor and uneven drainage. Routing of surface water through drainage networks in subarctic regions is similarly complicated by permafrost soils (McNamara and others 1999); (Boggart and others 2003); (Luoto 2007), which strongly influence watershed runoff processes (Jones and others 2005); (Jones and Rineart 2010) and thus downstream flow regimes, water quality, and aquatic habitat (Gomi and others 2002); (Freeman and others 2007). Over long periods, headwater channels and drainage networks respond to changing climate and tectonics, whereas short-term responses can be caused by human land-use (Montgomery and Dietrich 1989); (Dietrich and Dunne 1993); (Knighton 1998). In wildland watersheds, land uses potentially impacting soil erosion, channel initiation, and runoff patterns typically relate to resource extraction or recreational activities and associated road or trail networks (Montgomery 1994). Disturbances resulting from such activities can impact watershed processes by modifying both surface and subsurface flowpaths, resulting in the expansion of source areas during runoff events (Knighton 1998); (Winter 2007) and potentially causing greater fluxes of water, sediment, and nutrients downstream with consequent effects on riparian and benthic habitat (Freeman and others 2007); (Wipfli and others 2007). Such effects have been well documented in steep forested catchments in response to logging and roads (Montgomery 1994); (Jones and Grant 1996); (MacDonald and others 2004). Less well understood is how low-relief wildland watersheds respond to land-use impacts, particularly wetland-dominated landscapes where sediment production is low and drainage networks are poorly developed. In northern lowland landscapes, discontinuous or sporadic permafrost is already susceptible to a variety of natural thermokarst processes because of warm permafrost temperatures and variable ice-content (Jorgenson and Osterkamp 2005), which further complicates drainage network evolution and makes land surfaces more sensitive to local land-use disturbances.

The dominance of permafrost and associated lakes and wetlands at high latitudes (Smith and others 2007) and how these elements are responding to climate change is of increasing interest to resource managers (Rouse and others 1997); (Palmer and others 2009). A number of field and modeling studies have described complex interactions between permafrost degradation and the expression and behavior of hydrological processes in boreal settings due to warming temperatures, greater snowpacks, and the effects of fire and vegetation succession (Jones and others 2005); (Jorgenson and Osterkamp 2005); (Osterkamp and others 2009). Where land disturbances occur in zones with permafrost soils, resource managers are left with the question of whether watershed responses are due to local impacts only, a response to climate forcing, or some interaction between these drivers. Making decisions in light of such uncertainty is a source of major concern facing resource managers and policy makers in areas already subject to rapid climate change responses (Baron and others 2009), such as interior Alaska. In the many Alaska lands managed as parks (221, 294 km^2^ or 14.5% of the state), where extraction and industrial activities are typically prohibited, land-use concerns increasingly relate to subsistence and recreational activities, and the corresponding transportation infrastructure.

Increasingly summer travel away from the sparse road systems in interior Alaska is by off-road vehicles (ORVs), the majority of which are Class I ORVs that weight less than 360 kg with 4–6 low-pressure tires. Trends in this mode of transportation and recreation for Alaska follow those documented in many wildland areas throughout the U.S. and other countries (Cordell 2004), although in Alaska a high proportion of such activities are classified as subsistence use based on traditional uses by Native Alaskans and homesteaders (Slaughter and others 1990); (Happe and others 1998). As part of the Alaska National Interest Lands Conservation Act (ANILCA), a number of National Parks and Preserves were designated in Alaska with the stipulation that traditional resource uses be protected, including access to inholdings and other lands within them, and the continued subsistence harvest of fish and game (Willis 1985). Protection of these activities can potentially conflict with the historical National Park Service mandate “to conserve the scenery and the natural and historic objects and the wild life there in and to provide the enjoyment of the same in such a manner and by means as will leave them unimpaired for future generations” (National Park Service Organic Act, 16 USC1.). Such is the case in Wrangell-St. Elias National Park and Preserve (WRST), created in 1980 through ANILCA, where these lands were traditionally used for subsistence purposes prior to its creation and such use continues today with modern ORVs (Happe and others 1998). Recreational ORV use was also permitted on many traditional trail systems in WRST until recently when notable resource damage forced seasonal closure of six of the nine trails that were open to non-subsistence users (Jensen 2009). Besides the importance of WRST as an International World Heritage Site (World-Heritage-Committee 1994), the northwestern portion of the park forms the headwaters of the Copper River—arguably supporting one of the more important salmon fisheries in the world. A substantial number of known and probable salmon spawning and rearing lakes and tributaries occur within the area of actively used trails.

Localized impacts of ORV trails on soils and vegetation have been documented in WRST (Racine and Ahlstrand 1991); (Ahlstrand and Racine 1993); (Happe and others 1998) and other environments in Alaska (Rickard and Brown 1974); (Sparrow and others 1978); (Rinella and Bogan 2003). However, the degree to which ecosystem, watershed, and landscape functioning may be affected by these trails and the varying levels of use they experience is not well understood. Even less is known about how such impacts may interact with responses to ongoing and rapid climate change. It is recognized that trail impacts in WRST and similar ecosystems are most severe on lands with organic wetland soils, while watersheds with mineral soils and colluvial channels are more resilient (Slaughter and others 1990); (Happe and others 1998). In low relief, poorly drained areas of WRST with organic soils atop permafrost, heavily used trails tend to become braided as use intensifies (Happe and others 1998). Trail braiding occurs when multiple tracks diverge from and converge with the original trail in areas where it is less passable due to deep rutting and ponding of water. Locations of trail braiding often occur at stream crossings or other distinct points of flow concentration, such as hollows or groundwater-fed wetlands that are more prone to soil erosion and permafrost degradation, potentially initiating surface-water channels susceptible to headward erosion and the local expansion of drainage density (Dietrich and Dunne 1993); (Knighton 1998).

We hypothesized that trail braiding occurs more frequently at and upslope of locations where runoff accumulates, such as hollows and zones of groundwater discharge. The intersection of trails at these locations initiates or exacerbates upslope channel formation, thus creating a feedback between trail braiding and channel expansion. If this feedback occurs in low relief landscapes with organic, permafrost soils, such as portions of WRST, we would expect to see expansive zones of trail braiding upslope of channel initiation points and soil and runoff responses that promote further headward expansion. We evaluated this hypothesis by mapping current drainage patterns and comparing them to changes in ORV trail positions over the last half century. Additionally, we collected field data on soil temperature and runoff processes in order to determine if and to what extent trails altered the behavior of permafrost soils, rainfall-runoff responses, and erosion potential, which could allow this feedback between trail migration and channel headward expansion to occur. These regimes and responses were compared to an adjacent reference area with no trail access, but in a similar hydrogeomorphic setting and with an obvious point of natural channel initiation. Addressing this hypothesis is intended to better inform land managers seeking to understand the broader landscape consequences of local land-use disturbances, such as ORV trails, particularly as they concurrently plan for watershed responses to climate change in northern regions with permafrost soils.

## Study Area

**Fig. 1:**
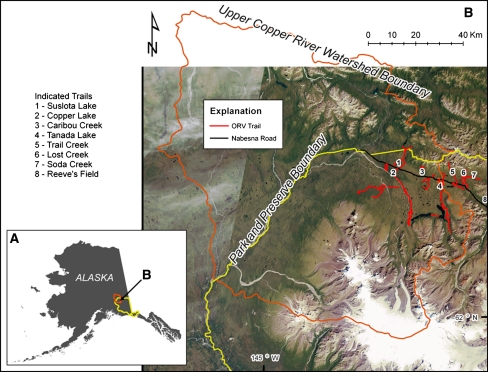
The location of the upper Copper River Watershed and boundary of Wrangell-St. Elias National Park and Preserve (WRST) in Alaska (**a**) and map of the NW portion of WRST with ORV trails and study area indicated (**b**)

The study area is located in the northwestern portion of WRST in eastern interior Alaska (Fig. 1). Access to this area is from the Glenn Highway at Slana via the 72 km Nabesna Road, from which ORV trails emanate (Fig. 1; Table 1). Vehicle travel along the Nabesna Road began in the early 20th century, primarily by miners and prospectors, and during the 1950s–1970s the road was improved to support seismic and mining activities (Happe and others 1998). Settlement by homesteading of this area occurred sporadically during this period with travel and trail use for subsistence hunting, fishing, and trapping. Following establishment of WRST in 1980, subsistence activities were allowed to continue under ANILCA (Willis 1985) and recreational activities along this corridor gradually increased (Happe and others 1998).

**Table 1:** Major ORV trail systems in NW Wrangell-St. Elias National Park and preserve including our focus study segment

Trail	Length (km)	Mean gradient (%)	Marked stream crossings	ORV trips (year^−1^)	Dominant land cover type
Suslota lake	13	0.7		60	Lowland black spruce forest
Copper lake	23	0.7	8	125	Riverine white spruce forest
Caribou creek	7	6.4	1	120	Subalpine willow—birch shrub
Tanada lake	25	0.3	18	65	Lowland black spruce forest
Study segment	2	0.9	2		Tussock-shrub bog
Reference area	–	–	–		Tussock-shrub bog
Trail creek	8	3.0	5	155	Subalpine spruce woodland
Lost creek	8	3.1	6	154	Subalpine willow—birch scrub
Soda creek	21	0.9	14	88	Subalpine willow—birch scrub
Reeve’s field	9	2.9	3	45	Riverine white spruce Forest

Mean gradient and blue line stream crossings are from 1:63,000 USGS topographic maps; ORV trips are round trips average per year from 1995 to 2005 from Nabesna ORV EIS, draft 2010, dominant landcover type is from Jorgenson and others (2008)

The Nabesna Road initially follows the upper Copper River valley, then crosses a low divide (990 m elevation) that separates the Copper River Basin from the Yukon River Basin. The road and river valleys are bounded by the Mentasta Mountains to the north and Wrangell Mountains to the south. Several relatively steep braided streams originating in the Mentasta Mountains cross the Nabesna Road from the north and provide easy access routes for ORVs. The dominant boreal ecotype along these trails changes from lowland black spruce forest to subalpine spruce woodland and willow and birch scrubland (Jorgenson and others 2008). Trails heading south off the Nabesna Road lead to large lakes at the Copper River headwaters, and are popular for providing access to prime hunting terrain in the Wrangell Mountains, primarily for Dall’s sheep (*Ovis dalli*). Boreal ecotypes in these intermountain lowlands are primarily black spruce forest and bog (Plant Association: *Picea mariana—Salix pulchra—Rubus chamaerus*) and tussock-scrub bog (*Eriophorum vaginatum—Betula nana*) with white spruce forests (*Picea glauca—Vaccinium uliginosum*) along alluvial terraces (Jorgenson and others 2008). The focus ORV trail for this study, the Tanada Lake Trail, traverses these intermountain lowlands. The reference site is located in nearby terrain with similar vegetation, soils, and hydrology, but without any ORV trails. The study section trail traverses a gentle slope, 1.4%, and the reference area is slightly steeper, 1.9%.

This intermountain lowland region of WRST was glaciated during the late Wisconsin (Last Glacial Maximum, ca. 20,000 years bp) (Manley and Kaufman 2002) and our study trail is covered with glacial moraines and drift and features that appear to be kames—mounds of layered sands and gravels often deposited at stagnating glacier margins. Soils are generally classified as discontinuous permafrost, but are near the boundary of continuous permafrost (Brown and others 2001), and are considered ice-rich in the mineral layers below organic horizons (*histic pergelic cyraquepts*). There is some observed polygonized tundra in this region though no evidence of ice-wedge networks nor frost scars occurs immediately along the study trail (Racine and Ahlstrand 1991). Interpolated long-term (1961–1990) climate records for the study area suggest mean annual precipitation of 740 mm and mean summer precipitation of 240 mm with a mean annual temperature of −7°C and a mean summer temperature of 10°C (PRISM Climate Group 2004). Data we collected in 2008–2009 from near Tanada Lake Trail trailhead showed slightly warmer and drier conditions with a mean annual temperature of −3.6°C, a mean summer temperature of 11.6°C, and summer precipitation of 205 mm.

## Methods

### Field Mapping and Aerial Photography Analysis

In June and September 2009, we mapped the current location and condition of ORV trails along a 2 km straight-line segment of the Tanada Lake Trail beginning at the trailhead located at mile 24.0 of Nabesna Road. At approximately 100 m intervals, we recorded the position of all identifiable secondary trails along a transect perpendicular to the primary trail. At these same locations, we recorded observations of surface soil type (mineral or organic), trail cover (bare or vegetated), and the presence or absence of ponded water. Additionally, we mapped all stream crossing points and the boundaries of distinct soil / vegetation patches traversed by the main trail network. To assess historic changes in trail position, course, and extent along this 2 km study section, we acquired aerial photography covering the entire Tanada Lake Trail and reference study area. This included black and white imagery from 24-Aug-1957 (1:21,000), color infrared imagery (CIR) from 7-Aug-1981 (1:65,000), and CIR imagery from 3-Aug-2004 (1:40,000). Aerial photographs were mosaicked, orthorectified, and georeferenced to 2005 IKONOS imagery, and then re-sampled to a 1-meter resolution in a geographic information system (GIS) database. Trails were manually delineated in each image at a 1:3,000 resolution and compared to ground observations made in September 2009. Nearly all trails identified in imagery from 1957, 1981, and 2004 could be located in the field. Differences in the course, position, and extent were compared among time periods to understand how the trails had changed over time, particularly with respect to current stream crossing points and locations of channel initiation.

### Soil Monitoring

**Fig. 2:**
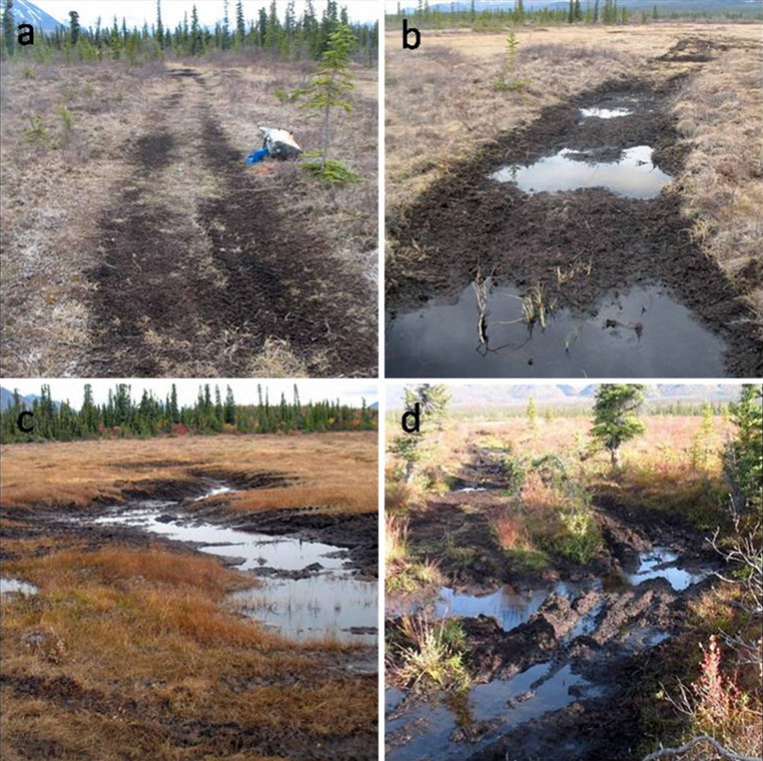
Examples of our focus study segment on Tanada Lake Trail showing (**a**) partly vegetated trail on mineral soil (soil monitoring station STT-6), **b** unvegetated trail on organic soils with ponding (soil monitoring station STT-3), **c** trail stream crossing (TC-4) and channel initiation point with mineral soil and partly underlain by till, and **d** trail stream crossing (TC-3) and channel initiation point with organic soil and underlain by shallow permafrost

To understand how and to what extent ORVs influenced soil temperature regimes, we monitored ground temperatures for one year at 10- and 50-cm depth to characterize freeze and thaw conditions near the surface and near the base of the active layer, respectively. We selected three types of trail conditions in which to monitor soil temperature, with two replications of each type: (1) dry mineral soil, (2) dry organic soil, and (3) saturated (ponded) organic soil (Fig. 2). We hypothesized that the last type would show the greatest deviation from reference conditions. At each trail site, we installed temperature sensor pairs (Onset Model TMC6-HD, ±0.2°C) directly into trail centers and 1 m from the trail edge. Trail center and edge sensor pairs were set to log temperature at hourly intervals using 4-channel external data loggers (Onset U12). Data collections spanned the period from 2-Sept-2008 to 10-Sept-2009. In addition to the in-trail and trail-proximal sites, sensors were installed at two sites approximately 50 m up-gradient and 50 m down-gradient from the trail course to serve as references. Instruments were checked and data were downloaded in late June and again in early September 2009. Each time data were downloaded, four active-layer depth (ALD) measurements were made as depth-to-refusal with a steel probe at 50 cm distance from the sensor, one in each cardinal direction, in order to compare ALD progression among monitoring sites.

To make more spatially integrated measurements of ALD and to begin a baseline for future monitoring of the seasonally thawed layer, we established two 1 ha (121 point) active-layer grids using protocols developed for the Circumpolar Active Layer Monitoring program (CALM, http://www.udel.edu/Geography/calm/); one grid at the reference site (designated as the Mentasta Fen site) and one at a site dissected by braided ORV trails (designated the Tanada Muskeg site). Both CALM grids were instrumented with soil temperature sensors at depth of 10, 50, 100, and 150 cm and set to log at 2 h intervals year-round. A full description of this method is provided in (Nelson and Hinkel 2003) and a brief description follows. A 100 × 100 m grid was laid out using an auto-level and tape with corners of the grid marked and geo-located. At 10 m intervals, ALD was measured with a steel probe to ±0.5 cm. ALD measurements were recorded on 10-September-2009 and again on 16-September-2010, dates when seasonal thaw should be near its maximum (Nelson and Hinkel 2003). Soil temperature sensors were serviced and data downloaded at this time. Differences in ALD between CALM grids were compared statistically with a student’s *T* test using an α of 0.05 to indicate a significant difference in means.

### Stream Crossing Monitoring and Analysis

Within the 2 km Tanada Lake Trail study segment, we identified locations where the trail crossed a stream channel as well as a nearby reference site where a channel initiated from below a small peatland at a similar landscape position as trail crossings (Fig. 2). For each channel, we surveyed ground and water surface, and ALD or depth to till or other coarse rocky layer, using a total station (Nikon DTM-352) and prism rod, with an emphasis on measuring the longitudinal and cross-sectional form of each channel from above the point of channel initiation, across the trail, and up to 100 m downstream. These measurements were used to characterize the channel form, water surface slope, and channel depth distribution at each site. To monitor water depth, we anchored pressure transducers (Onset, U20 ± 0.5 cm) to the channel bed in deeper pool locations approximately 10–20 m upstream and 10–20 m downstream of trail crossings. Pressure readings at 15 min intervals were compared to barometric pressure measurements made at the weather station (within 2 km of all channels) to calculate stream depths during the ice-free season. The one exception was at the reference site 20 m upstream of the channel initiation point, where we instead installed a shallow well (0.6 m depth) cased and fully screened with machine-slotted PVC (5.1 cm diameter) with a pressure transducer suspended from a nylon cord 20 cm below the soil surface. Water depth records were used to estimate flowing water periods based on comparisons with survey data and to measure any changes in water surface slope across trail crossings. These hydraulic measurements were used to estimate boundary shear stress (τ_o_) at hourly intervals for each trail crossing and the reference site, as 1;

1 $$ \tau_{\text{o}} = \rho_{\text{w}} gds $$

where ρ_w_ is the density of water, *g* is gravitational acceleration, *d* is the mean water depth, and *s* is the water surface slope. We compared τ_o_ to critical shear stress (τ_cr_) estimated from the Shield’s equation for channels with beds ranging from silt to cobble, as 2;

2 $$ \tau_{\text{cr}} = kg\left( {\rho_{\text{s}} - \rho_{\text{w}} } \right)D $$

where *k* is the critical dimensionless shear stress (set at 0.045), ρ_s_ is sediment density (set at 2,650 kg/m^3^), and *D* is the sediment diameter (Knighton 1998). For peat channels, we estimated τ_cr_ from reported values of shear strength (σ_s_) in the literature using the empirical relation 3;

3 $$ \tau_{\text{cr}} = 1.3\sigma_{\text{s}} + 3.6 $$

as reported by Knapen and Poesen (2010). Values of τ_o_ > τ_cr_ suggest sediment mobility and erosion of the channel bed, though it is recognized that these are very approximate estimates. The role of thermal erosion of permafrost soils was not accounted for in this study.

## Results

### Patterns of Trail Development

For context, there are seven major ORV trail systems spanning 114 km that can be accessed from the Nabesna Road in WRST (Fig. 1; Table 1). These trails can generally be classified as either (1) following steeper valleys of mountain streams and adjacent hillslopes (Trail Creek, Lost Creek, and Soda Creek), (2) following river floodplains (Copper Lake and Reeve’s Field), or (3) traversing intermountain lowlands (Tanada Lake and Suslota Lake). We focused on this last category of trail (those dominated by lowland black spruce forest with slight gradients) because previous work from WRST and other regions have shown that these ecosystems are most prone to impacts to vegetation and soils (Sparrow and others 1978); (Racine and Ahlstrand 1991). Approximately a third of active ORV trails along the Nabesna Road corridor are in this category. Our study focused on a 2 km segment of the Tanada Lake Trail, which had moderate ORV use during the period of NPS monitoring (Table 1), but much of this travel occurs in the late summer for sheep hunting, when soils are most prone to degradation .

**Fig. 3:**
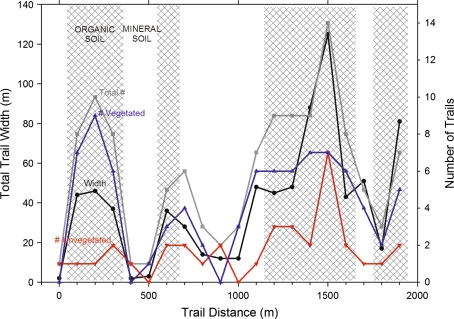
Study segments characteristics along the Tanada Lake Trail sampled at 100 m increments

The study segment of the Tanada Lake Trail showed active and past erosion, thaw-subsidence, and braiding, with the extent of damage varying substantially depending on trail use, soil type, and associated vegetation. Over a 2 km straight-line distance, the trail crosses eight distinct soil-vegetation patches (Fig. 3); 35% of the length consisted of mineral soils supporting primarily white spruce forest, spike-rush meadows, or mixed grass-shrub meadows and the other 65% consisted of organic soils supporting black spruce muskeg, sedge fen, or tussocks-scrub bog. Sections of trail crossing mineral soils were typically single-thread tracks with largest amount of braiding in a dry meadow area where five semi-parallel tracks were observed. No notable ponding of water was observed in these sections during the survey conducted in early September 2009. Additionally, more than half of trails crossing mineral soil had some vegetative cover. Conversely, sections of trail crossing organic soils often had extensive braiding with an average of 8 semi-parallel tracks covering a width of 17–125 m, and up to 14 individual trails in one location (Fig. 3). About 25% of trail braids on organic soils were unvegetated, while the rest supported some vegetation on newly-formed or infrequently-used trails or had re-vegetated following inactivity. In addition, these areas were characterized by the presence of ponded water in trail depressions resulting from a combination of erosion and thaw-subsidence. Most notable was that more severely degraded trails were consistently found along the upslope edge (east side) of the trail corridor and also were more recent, based on an examination of the repeat aerial photography. Two perennial streams and at least two ephemeral channels were crossed by the trail and it was also at these locations that trail braiding, thaw-subsidence, and soil erosion appeared most severe.

**Fig. 4:**
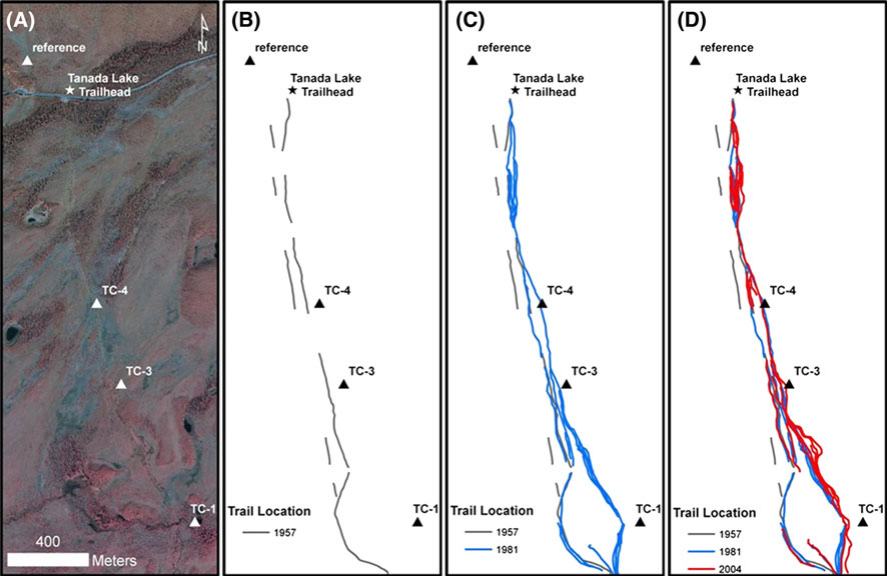
View of the Tanada Lake Trail study segment from 2004 color-infrared photography with channel initiation points indicated (**a**) and location of ORV trails observed in 1957 (**b**), 1981 (**c**), and 2004 (**d**)

**Fig. 5:**
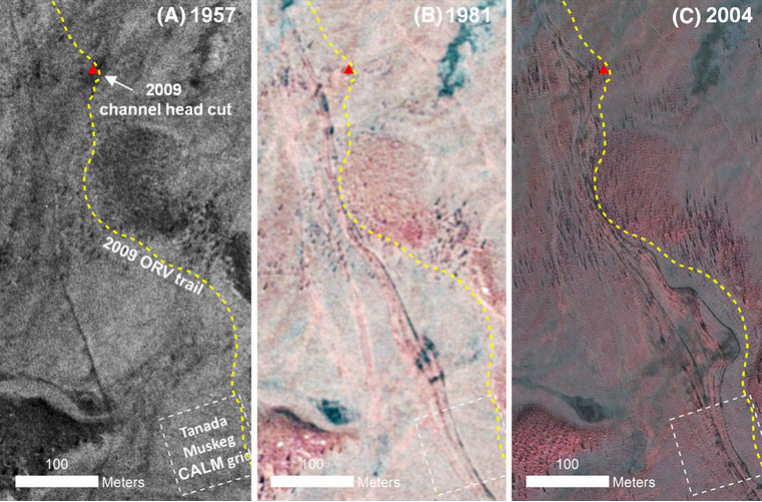
Time series of aerial photographs in **a** 1957, **b** 1981, and **c** 2004 for a portion of the Tanada Lake Trail study segment at the resolution used for identification of trails. The location of the channel head at TC-3 and the contemporary ORV trail in 2009 are indicated for reference

To analyze changes in the Tanada Lake Trail network during the last half-century, we mapped the trail course from 1 m resolution aerial photography acquired during the summers of 1957, 1981, and 2004 and compared these images to points mapped on the ground in 2009 (Fig. 4). In 1957, 23 years before WRST was established, the Tanada Lake Trail began at the contemporary location (although no trailhead pullout was yet present) and a single, relatively straight track could be distinguished with a very intermittent, parallel trail observed about 50 m downslope (Fig. 4b). Whether this track represents a much earlier abandoned trail or a contemporary less frequently used trail is uncertain. The total length of distinguishable trails in 1957 was 2,810 m, or 1.2× the straight-line distance. The major deviation in the 1957 course from a straight-line-of-travel occurred at the first perennial stream crossing (TC-1) at about 350 m downstream from the current crossing point (Fig. 4b). Though the ephemeral channels TC-3 and TC-4 could not be recognized in 1957, the location of the trail was 85 and 65 m, respectively, downstream of the 2009 mapped channel initiation points. By 1981, the year after WRST was designated as national parkland, the trail network had expanded by 180% to 3.3× the straight-line distance, with trails following the 1957 route and also a newer set of upslope routes crossing TC-1 near the 2009 location (Fig. 4c). The 1981 trail crosses TC-4 at the 2009 location and point of channel initiation and crosses TC-3 40 m downstream of the 2009 channel initiation point. In 2004, the trail system continued to advance upslope with more intense braiding; however, much of the original downslope trail observed in 1957 could no longer be seen in 2004 imagery, such that total trail length remained stable relative to 1981 at 7,890 m (Fig. 4d). The most pronounced shifts between 1981 and 2004 occurred (1) in tussock-scrub bog near the trailhead, where 6–7 trail braids were apparent, (2) at TC-3 where four trail braids were noted with the upslope track within 20 m of the 2009 channel initiation point (Fig. 5), and (3) in a wide peatland between TC-3 and TC-1 where seven braids were noted and were now positioned 55 m upslope of the 1981 trail and 160 m upslope of the 1957 trail (Figs. 4d, 5). Analysis of the reference site over this 47 year period showed no evidence of ORV trails and the channel initiation point remained stable.

### Soil Temperature Regimes and Active-layer Thickness

In order to understand how trail networks affected freeze and thaw of soils of differing edaphic and hydrologic condition, six monitoring stations were established at trail sites along with two stations at reference sites. At the reference stations, located above and below active trails, the ALD as measured in September ranged from 64 to 75 cm. The soil froze at 50 cm depth in early October 2008 and thawed in late July 2009 with a mean annual ground temperature (MAGT) of −1.5°C. The mean winter ground temperature (MWGT) at 50 cm was −2.4°C, and the mean summer ground temperature (MSGT) at 50 cm was just above freezing, 0.1°C (Table 2). For trails crossing mineral soils, the September ALD averaged 92 cm at trail centers and 46 cm at adjacent trail margins, with soils at both locations freezing around mid-November 2008 at 50 cm depth, although the trail center location thawed much earlier (6-Jun-2009) than the margin (27-Jul-2009). MAGT measured at 50 cm depth for trails on mineral soils was similar in the trail centers vs. the margin (−1.3°C), but soil temperature at the trail center was much colder in the winter and warmer in the summer relative to the trail margin where vegetation cover was mostly intact (Table 2). On organic soils, trails could be divided into locations with major thaw-subsidence and erosion forming pools and areas of denuded vegetation only without standing water. For all trails with organic soils, the September ALD was greater at trail centers (71–82 cm) compared to trail margins (49–59 cm). Trail centers at ponded sites froze earlier and thawed earlier than those at areas of dry trail, whereas the trail margins of both types were relatively similar (Table 2). Temperature regimes at ponded trail centers, as represented by MAGT, MWGT, and MSGT, demonstrate that soils in these areas were warmer throughout the year compared to dry organic trail centers and the trail margins of both organic trail types.

**Table 2:** Soil monitoring stations characteristics and regimes from 2008 to 09

Site	June ALD (cm)	Max ALD (cm)	Freeze date	Thaw date	MAGT (C)	MWGT (C)	MSGT (C)
Reference							
Above trail	28	75	Oct-2	Jul-27	−1.9	−2.8	0.0
Below trail	24	64	Oct-6	Jul-30	−1.2	−2.2	0.1
Mineral							
Trail center	45	92	Nov-13	Jun-6	−1.0	−6.2	3.8
Trail side	30	45	Nov-12	July-27	−1.5	−3.9	0.2
Organic (dry)							
Trail center	38	71	Nov-2	Jul-3	−2.0	−5.0	1.4
Trail side	30	48	Oct-14	Aug-15	−2.9	−6.2	−0.3
Organic (ponded)							
Trail center	64	82	Oct-8	Jun-22	−0.3	−2.7	2.3
Trail side	31	58	Oct-29	Jul-18	−2.4	−5.6	0.5

Reference sites are from individual stations, while mineral, dry and pooled organic sites are means from two stations

*ALD* active-layer depth, *MAGT* mean annual ground temperature, *MWGT* mean winter ground temperature, *MSGT* mean summer ground temperature at 50 cm depth

**Fig. 6:**
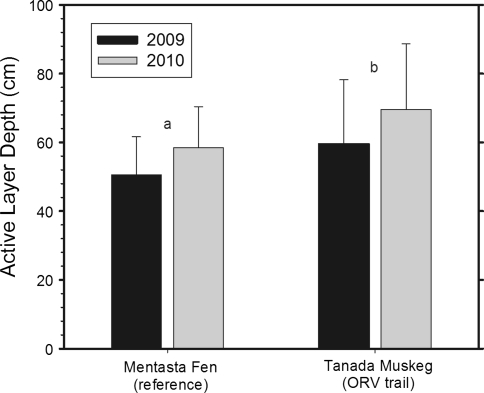
Mean active-layer depths from 121 point, 1 ha grids measured on 10-Sept-2009 and 16-Sept-2010 at a reference site with no ORV trails and at a site with substantial ORV trails and braiding. Error bars are standard deviations and letter indicate a significant difference at *P* < 0.01 using a student’s *T* test between sites for both years

**Fig. 7:**
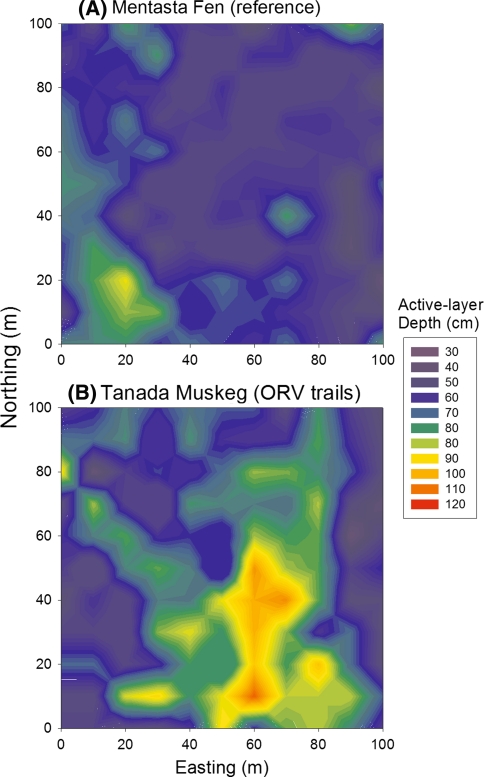
Spatially distributed active-layer depths measured at 1 ha study grids on 10-Sept-2009 at 10 m intervals and interpolated to 1 m raster grid

A more spatially comprehensive assessment of ALD was made using CALM grids (100 × 100 m, 121 points) at the reference site (Mentasta Fen) and at a highly disturbed trail site (Tanada Lake Muskeg) that is bisected by a swath of trail braids. Both sites are moderately sloping (1–2%) with organic soils supporting tussock peatland interspersed with black spruce muskeg. Comparison of these CALM grids showed a significantly deeper mean ALD of 59.5 cm (±18.7 cm SD) at the trail site compared to a mean ALD of 50.5 cm (±11.1 cm SD) at the reference site (*P* < 0.01) (Fig. 6). At the reference site, variation in ALD was relatively moderate, ranging from 30 to 85 cm, whereas at the trail site there was a zone of deep thaw >100 cm that corresponded to a set of trails with deep thaw subsidence (Fig. 7). In September 2010, the mean ALD was deeper at both CALM sites, 58.5 cm (±11.9 cm SD) at the Mentasta Fen reference site and 69.6 cm (±19.1 cm SD) at the Tanada Lake Muskeg disturbed site, likely due to a slightly warmer and wetter summer than in 2009 (Fig. 6).

### Channel Initiation Points and Stream Crossings

**Fig. 8:**
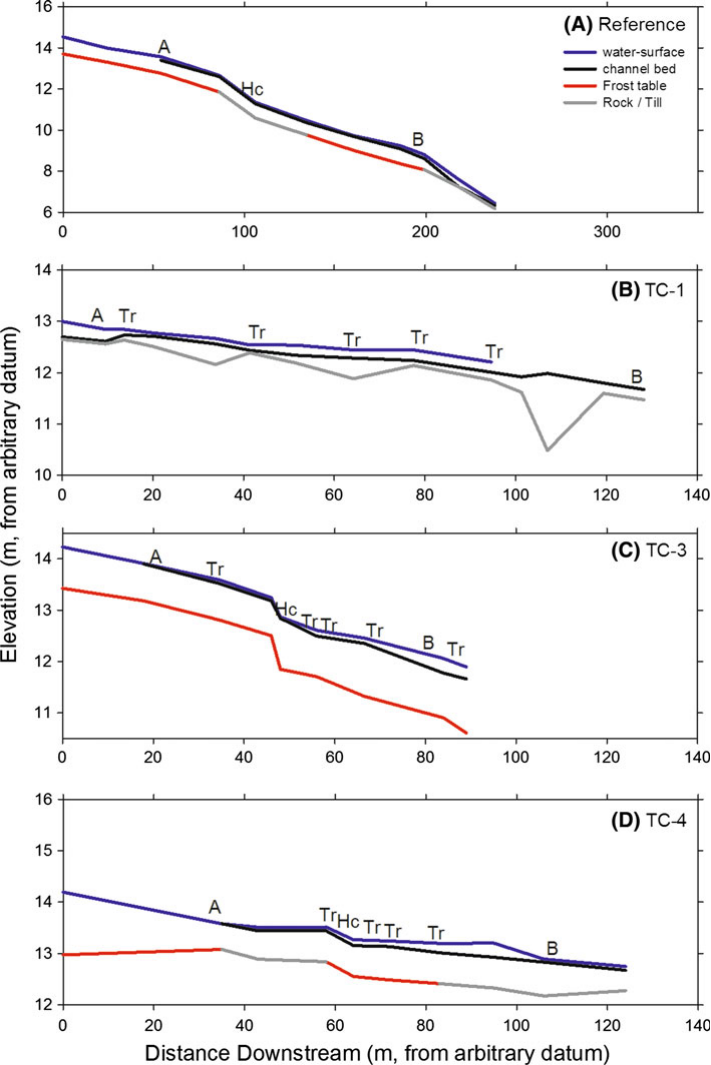
Longitudinal profiles of the reference study site and three reaches crossed by trails surveyed in early September 2009 at approximately 10–15 points per reach including measurements of depth-to-refusal that were differentiated as frozen ground or rock (sand to cobble/coarse till) (*A* location of the above trail, *B* location of the below trail monitoring stations, *Hc* location identified channel initiation points, and *Tr* location of trail crossings of the stream channel)

To understand how streams and their channels have been impacted by trail crossing points, we analyzed the form and behavior of three channels intersected by the Tanada Lake Trail and compared these to a nearby reference site stream. The reference stream initiates from the toe of a fen set in a small basin, transitioning from a peat body with a steepening channel, 2.3%, that cuts down to armored till up to large-cobble size (Table 3). A distinct headcut is indicated in Fig. 8a where the channel steepens to >6% over a short distance. The largest trail-crossed stream, TC-1, initiates from a pond above the trail system and has a shallow gradient, 1.1%, and a channel composed of cobble transitioning into gravel below the active trail crossing. TC-3 initiates from a tussock fen transitioning into black spruce muskeg with an average slope of 2.0% and a steep headcut dropping >15% over a 2 m channel distance (Figs. 8c, 2d). As a result of the intense trail use it is difficult to tell if this was a natural transition or whether it was due to erosion and thaw subsidence. The entire channel of TC-3 is composed of organic soils and peat, stabilized to some degree by spruce and willow roots. The TC-4 channel initiates at a transition between organic and mineral soils, is mainly composed of silt and sand underlain by coarse till and an obvious, but moderate, headcut with a 4% slope over 3 m (Figs. 8d; 2c).

**Table 3:** Stream characteristics and summer 2009 regimes for monitoring stations above and below trail crossings (TC sites) and a natural channel initiation point (Ref site)

Station	Width (m)	Depth (m)	Slope (%)	Bed substrate	Summer flow durations (%)			Peak depth (m)
					Dry	Baseflow	Floodflow	
Reference								
A—CIP	–	–	1.2	Peat	–	–	–	0.25
B—CIP	0.4	0.51	2.3	Cobble	10.5	74.5	15.0	0.96
Trail crossing-1 (TC-1)								
A—trail	0.8	0.27	1.1	Cobble	38.5	52.9	8.6	0.40
B—trail	1.3	0.24	1.1	Gravel	69.6	11.7	7.7	0.65
Trail crossing-3 (TC-3)								
A—trail	0.4	0.14	1.9	Peat	0.2	93.1	6.7	0.18
B—trail	4.5	0.28	2.0	Peat	24.9	71.9	3.2	0.30
Trail crossing-4 (TC-4)								
A—trail	1.9	0.14	1.6	Silt	0.2	99.6	0.2	0.15
B—trail	3.8	0.11	1.5	Silt	13.1	83.6	3.3	0.13

Channel width and depth are for local full capacity from single cross-sections surveys and slope is measured over at 10–30 m stream distance. Flood flow was defined as depths greater than channel capacity and base flow was defined as below channel capacity

Water depth in each channel was monitored upstream and downstream of trail crossing locations to understand temporal shifts in streamflow regime and energy gradients across trail crossings during the spring and summer of 2009. At the reference site, water levels were measured in a shallow well above the channel initiation point and approximately mid-way to the downstream location in an area where ORV traffic would most likely cross if travel were to occur through this area. Particular emphasis was placed on assessing flow responses to rainfall events, of which there were seven that surpassed 10 mm/d, accounting for 62% of the 205 mm precipitation measured between 12-May- and 7-Sept-2009. During this period, streams TC-3 and TC-4 above the trail crossing showed perennial flow with varying durations of baseflow or floodflow, while at TC-1 below the trail crossing flow was intermittent with a dry channel for >70% of this period. Intermittent streamflow was observed more frequently below trail crossings where the majority of channels surveyed were wider and more shallow below the trail than in upstream channels (Table 3).

**Fig. 9:**
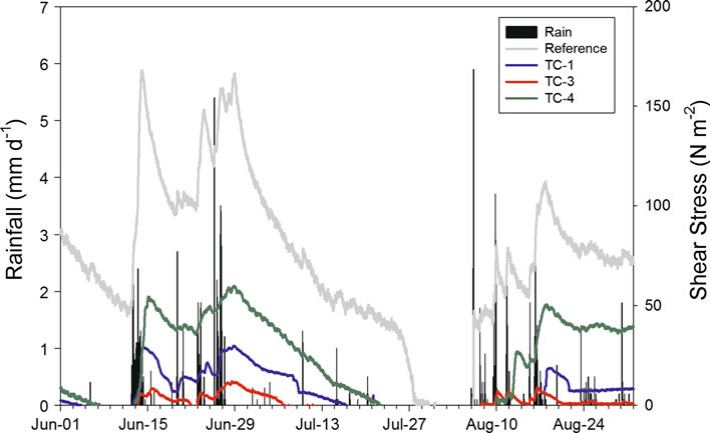
A comparison of rainfall patterns in relation to variation in estimated shear stress regimes at each stream each (channel initiation point of reference reach and stream crossings)

An estimate of the shear stress (τ_o_) generated by streamflow between depth monitoring stations throughout the spring and summer is reported in Fig. 9. Here, shear stress dynamics are a function of change in water depth and variation in the water-surface slope, with values approaching critical shear stress (τ_cr_) during several rainfall-runoff events that occurred in mid- to late-June and again in early- to mid-August. The higher values occurring in June are likely because the ALD’s early in the summer are shallow and therefore runoff responses are expected to be more rapid. For example, the highest intensity rainfall event, 6 mm/hr on 6-Aug, had little impact on streamflow, probably because it followed a period of drought when streams were mainly dry, ALDs at this date were deeper, and soil-water storage was depleted. The highest τ_o_ values generated were at the reference site, exceeding 100 N/m^2^, where the energy gradient was steepest. The bed of the reference channel flows over till of cobble size, much of which appeared to be armored by this coarse sediment. The shear stress required to move such material potentially exceeds 190 N/m^2^ for cobble (Table 4) due to armoring, such that entrainment of the cobble / till bed at the reference site was unlikely during any of the rainfall events in 2009. At TC-3, maximum τ_o_ exceeded 65 N/m^2^ during several rain events in June and appeared to be primarily driven by increasing depth rather than changes in energy gradient across the trail zone, although stream slopes were more variable in this section of the channel due to a pit that formed at the trail crossing point, which resulted in ponding (Figs. 2d, 9). The resistance of peat channels to erosion, and the corresponding erosion thresholds, particularly in permafrost, are poorly understood or at least poorly documented. Our best estimates of τ_cr_ for peat soils range between 30 and 210 N/m^2^ (Cola and Cortellazzo 2005) also suggesting potential channel erosion and headward migration, though no evidence of these processes were actively observed in 2009. However, in the fall of 2010 after our monitoring stations were removed, more intense rainfall events occurred in this area and the point of channel initiation was observed to have migrated headward several meters. Estimated τ_o_ values at TC-1 and TC-3 were considerably lower, but still within potential thresholds for bed erosion and entrainment given that their channel substrates were primarily of gravel and silt, respectively. Unaccounted for in this analysis of the potential for channel erosion is the changing resistance of frozen soils during the summer and the role of thermal versus mechanical erosion. Soil temperature monitoring suggested, however, that at trail crossings, surface soils were thawed during the time period when significant rainfall-runoff events first commenced in 2009.

**Table 4:** Estimates of range for critical shear stress required for mobilization of channel substrate types found at study streams

Channel composition	Critical shear stress (*N*/m^2^)	Source
Cobble	45–190	Shields equation (*k* = 0.045)
Gravel	1.5–45	Shields Equation (*k* = 0.045)
Sand	2.5–3.5	Knapen and others 2007
Silt	2.5–4.5	Knapen and others 2007
Peat	30–210	Cola and Cortellazzo (2005)

## Discussion

Research on ORV trails in Alaskan boreal landscapes began in the 1970s when ORV use first became widespread and the sensitivity of permafrost terrain to damage was recognized (Slaughter and others 1990). Several studies in the arctic and subarctic demonstrated that trail impacts to vegetation communities and permafrost soils were pronounced in low relief areas with organic soils and poor drainage (Rickard and Brown 1974), whereas travel on upland well-drained soils or floodplains often resulted in minimal impacts (Sparrow and others 1978). Major impacts to lowlands include denudation of vegetation cover and organic horizons, an increase in active-layer depth, and ground subsidence or deflation (Lawson 1986); (Slaughter and others 1990); (Racine and Ahlstrand 1991). Such impacts are often associated with trail widening and braiding that greatly expands the zone of damage beyond a narrow travel corridor. Additionally, these studies generally showed lasting impacts once trails are abandoned or use restricted, suggesting either slow recovery or continued degradation following the initial disturbance (Sparrow and others 1978); (Happe and others 1998). Our research in WRST documents these same patterns in ORV trail braiding, vegetation denudation, organic layer destruction, and permafrost degradation with variations dependent on soil type, vegetation, and trail use across mosaics of tundra wetland and forest. Although much of the earlier work on ORV impacts in a typically boreal, lowland setting was also conducted in WRST, the emphasis was on understanding the damage created by industrial ORVs and the intensity and timing of their use (Racine and Ahlstrand 1991); (Ahlstrand and Racine 1993). However, off-road travel in the WRST area and elsewhere has progressively shifted towards more recreational activities and lighter, more versatile, ORVs. This shift resulted in an expanded area of impact once the park was created in 1980 (Happe and others 1998) that continues to the present day. While localized impacts have thus been documented, what remains unclear is whether such disturbances are manifested at larger scales in the form of alterations in watershed hydrology and downstream ecosystems.

The process of trail widening and braiding in relation to landscape drainage patterns and the location of channel heads is likely the key aspect determining whether the impacts of ORV trails remain confined to a narrow corridor of travel or can propagate upstream and downstream of the traversed hillslope to impact larger portions of the watershed. As trail use intensifies, denudation of vegetation and organic mats reduces insulation of permafrost soils, thereby causing erosion, thaw subsidence, and ponding of water. Ponded water further exacerbates subsidence, and trails can become impassable for most ORVs during wet conditions (Sparrow and others 1978); (Racine and Ahlstrand 1991); (Happe and others 1998). Once locations along a trail degrade to this condition, ORV users may deviate from straight-trail courses, creating new trails to one side or the other, thus forming trail braids. Particularly in locations where the water table is high, such as depressions or zones of flow convergence, this process of trail degradation, avoidance, and braiding may become quite pronounced. This pattern is easily recognized at several locations along the Tanada Lake Trail in WRST where a single-thread trail of <3 m width crosses from well-drained mineral soils to poorly-drained organic soils and quickly expands to >100 m in width with >5 individual tracks (Fig. 3).

A key observation from the present study is that braiding typically occurs upslope of the original degraded trail, such that trails progressively migrate upslope and may even cause the entire trail corridor to migrate upslope to create a new straighter trail as ORV users appear to seek a shorter, less sinuous line of travel. This is most evident in changes in trail routes between 1957 and 1981 along the study segment of the Tanada Lake Trail approaching TC-1 (Figs. 4, 5). Some of these trail degradation points are at streams crossings, where they impact the channel bed and may cause an increase in sediment flux downstream and channel adjustment both upstream and downstream of the channel crossing (Rinella and Bogan 2003). For channels with gravel to cobble beds, impacts at stream crossing are generally minor and localized, but for channels composed of silt or peat with additional stability provided by riparian vegetation, stream crossings become easily degraded and impacts can propagate upstream and downstream. It is at wetland crossings, however, where the more critical positive feedback is likely occurring between trail braiding and watershed processes. On gently sloping terrain, wetlands occur at broad hollows and points of flow convergence with organic soils and dense vegetation atop permafrost, both of which make soils resistant to initiation of channelized flow. ORVs traversing these wetlands remove the vegetation and organic soils, which then cause thaw subsidence and exacerbate flow convergence by creating a trough that intercepts the water table. During runoff events, when the water table rises above the soil surface, water that would naturally move as low velocity sheet flow through dense vegetation and tussocks now potentially forms rills and gullies over denuded, low resistance soils with greater energy to cause thermal and mechanical erosion of permafrost soils, particularly in areas with a shallow active layer. Continued braiding upslope of trail degradation points creates an expanding zone that routes ground-water flow to the surface with increasing erosive energy during runoff events—potentially initiating channels and locally expanding drainage networks. Our study documents that this set of positive feedbacks is occurring along the Tanada Lake Trail in WRST; specifically by upslope trail-braid migration at trail degradation points in combination with runoff responses sufficient to cause channel initiation, headward erosion, and thus local expansion of drainage networks. The rate of channel initiation and the number of headward erosion points in WRST remains uncertain; these are questions which future analysis with ground and aerial surveys at high resolution (i.e., photography and LiDAR) coupled with runoff-response monitoring should address.

The hydrogeomorphic conditions and source areas for channel initiation and the corresponding watershed drainage density are fundamental attributes of watershed behavior (Montgomery and Dietrich 1989); (Knighton 1998) that influence downstream flow regimes, export of sediment and nutrients, and benthic and riparian habitat (Gomi and others 2002); (Freeman and others 2007). In subarctic regions, permafrost soils exert first-order control on drainage density patterns and runoff processes and there is much interest in understanding how these processes may respond to observed and projected increases in permafrost degradation and changing active-layer depths and regimes (McNamara and others 1999); (Boggart and others 2003); (MacDonald and others 2006); (Luoto 2007). An important challenge for northern resource managers, particularly of boreal and arctic parklands, is understanding the potential landscape-scale responses to climate change and being able to separate such responses from the impacts of local activities, such as recreational and subsistence activities. Such an understanding is essential because the impacts of local activities can be managed directly through policy, regulation, and restoration (Baron and others 2009), whereas if for example wide-spread permafrost degradation is a function of increasing temperature and moisture regimes only (Jorgenson and Osterkamp 2005); (Osterkamp and others 2009), few management options are available to local resource management agencies. Of particular concern are situations in which local human disturbances interact with regional climate change responses to exacerbate these disturbances (Baron and others 2009), particularly those that impact watershed-scale processes and downstream water resources (Rouse and others 1997); (Palmer and others 2009).

## Conclusions and Recommendations

The parklands of WRST are a national public resource, a designated World Heritage Site, and form the headwaters of the Copper River watershed, and support spawning grounds for an economically important salmon fishery. Thus the importance of managing such park watersheds to promote natural functioning for maintaining landscape integrity and downstream habitat and water quality would seem evident. However, managing for multiple resources uses, such as recreational and subsistence trail use, particularly within the context under which WRST and other Alaska national parks were established, has created challenging circumstances for meeting traditional park mandates.

Localized damage to vegetation and soils due to ORV travel across certain ecotypes, specifically poorly drained organic soils atop permafrost, has been documented in WRST and other boreal landscapes, yet little is known about broader landscape-scale impacts of such disturbances. This study describes an important feedback between trail use and drainage systems where trails progressively migrate upslope of degraded stream crossings and points of channel initiation, thus expanding the drainage network and altering watershed functioning. We also document modifications in soil temperature, rainfall-runoff responses, and erosion potential at these points that would allow this feedback to occur and create upslope migration of channel heads. Most notable is the continued widening of trail networks through braiding during a 50 year period where travel routes became more braided in the most sensitive areas—organic wetland soils and stream crossings. The tendency for new trails to be formed upslope of stream crossings and wetlands is most problematic in terms of causing headward expansion of existing channels or premature channel initiation when crossing wetlands. More detailed and long-term study is required to determine the extent of such hydrogeomorphic processes and whether these result in detectable impacts to streamflow and downstream water quality and aquatic habitat.

Potentially the most pressing concern in managing resource damage due to ORV trails in WRST, and similar settings in Alaska and other boreal regions, is predicting the outcomes of the various policy decisions available. Previous work suggests that soil and vegetation recovery following cessation of ORV trail use can be very slow to non-existent, and thus the propagation of trail impacts at the watershed-scale may continue even if trail use is closed. Responses of permafrost soils to climate change are also causing unprecedented changes in boreal landscapes, such that land-use impacts may be difficult to separate from those created by regional-scale climate and landscape changes. The establishment of long-term monitoring programs focused on both natural and human disturbed sites will provide crucial data for use in separating localized disturbances from those due to climate change, particularly in lands managed as parks where long-term support for such monitoring efforts should be most available. As part of this study, we established standardized permafrost monitoring sites (CALM grids) at an ORV trail and a reference site with corresponding ground and air temperature monitoring to help assess interannual variability and long-term trends due to both climate and land use. A positive aspect of these trail disturbances in WRST is that an opportunity for restoration is now presented in which alternative designs or management strategies might be implemented with long-term monitoring programs developed to assess project success. Such a program would provide valuable information to better manage and restore similar lands in Alaska and other boreal landscapes subject to ORV trail impacts, which are increasingly widespread and difficult to regulate.
